# Optimization of control parameters of a hot cold controller by means of Simplex type methods

**DOI:** 10.1155/S1463924697000035

**Published:** 1997

**Authors:** C. Porte, M. Caron-Poussin, S. Carot, C. Couriol, M. Martin Moreno, A. Delacroix

**Affiliations:** Laboratoire de Chimie Industrielle CNAM 292 rue Saint Martin Paris Cédex 03 75141 France

## Abstract

This paper describes a hot/cold controller for regulating crystallization operations. The system was identified with a common method (the Broida method) and the parameters were obtained by the Ziegler-Nichols method. The paper shows that this empirical method will only allow a qualitative approach to regulation and that, in some instances, the parameters obtained are unreliable and therefore cannot be used to cancel variations between the set point and the actual values. Optimization methods were used to determine the regulation parameters and solve this identcation problem. It was found that the weighted centroid method was the best one.


Journal of Automatic Chemistry, Vol. 19, No. 1 (January–February 1997) pp. 15–26

### Optimization of control parameters of a hot cold controller by means of Simplex type methods

**C. Porte, M. Caron-Poussin, S. Carot, C. Couriol, M. Martin Moreno and A. Delacroix.**

*Laboratoire de Chimie Industrielle, CNAM, 292 rue Saint Martin, 75141 Paris Cédex 03, France*

*This paper describes a hot/cold controller for regulating crystallization operations. The system was identified with a common method (the Broida method) and the parameters were obtained by the Ziegler–Nichols method. The paper shows that this empirical method will only allow a qualitative approach to regulation and that, in some instances, the parameters obtained are unreliable and therefore cannot be used to cancel variations between the set point and the actual values. Optimization methods were used to determine the regulation parameters and solve this identification problem. It was found that the weighted centroid method was the best one.*

Until the 1970s, regulation, which is important in bulk chemistry, was based on traditional controllers: analogue, pneumatic or electrical. When microprocessors began to appear, numerical PID controllers were available and proved much easier to use and preset. In addition, controllers quickly became less expensive.

To maintain a temperature in chemistry, often involves complex regulation, either in cascade or of the hot/cold type. In both cases, the transfer functions of the systems have to be known so that the parameters involved in the regulation can be identified. This requires the use of empirical methods, such as the Broida method, which is associated with the Ziegler–Nichols method. These methods are easy to implement in terms of obtaining the parameters of the controller, but they require numerous 'tries' in order to reach the objective. This paper reports on the use of optimization methods to determine the optimal parameters of a hot/cold controller, which was being used for the regulation of the temperature in a reactor-crystallizer.

This paper describes the experimental set-up for a laboratory-scale crystallization apparatus and shows how the parameters for the PID controller were determined using the Broida and Ziegler–Nichols methods. The optimization of the settings are described, and the results obtained by different optimization methods are compared: simplex, modified simplex (Nelder and Mead), multi-move and weighted centroid method (WCM).

### The hot/cold controller

The aim was to regulate temperature control during a crystallization operation [1]. Temperature is controlled over time, so nucleation and growth rate can also be regulated. The control over temperature must be as accurate as possible and requires a good regulation around the desired value. This value varies over time and follows a polynomial law of the type $T = a + bt^3$, where $T$ is the temperature, $t$ the duration of the operation, and $a$ and $b$ are the parameters. The Pyromat 300 and Programat controllers, manufactured by Schlumberger, were used. The program emitter allows the set value to be changed over time.

The Pyromat 300 series are programmable PID numerical controllers. There is a channel dedicated to the cooling system and another dedicated to the heating system. The two channels are completely independent and can be set to analogue or numerical PID modes, They offer three possibilities for the heating and cooling channels: modulated/modulated, modulated/analogue, and analogue/modulated. The 'cold' and 'hot' channels can overlap for up to −10 to 20% of the command signal. Therefore, both the heating and cooling rate can be controlled.

### *The heating loop*

The heating loop consists of a Pt100 probe (TP) immersed in a 10 l thermostatted bath filled with water, with a circulation pump (flow rate 1000 l/h), a heating resistance (HR) of 1000 W connected to a power unit (PU) through a controller (C) (see figure 1).

The power unit is controlled by the analogue output (4–20 mA) of the 'hot' channel of the controller. This signal is converted by the power unit's interface to a percentage (0–100%) of the total heating power.

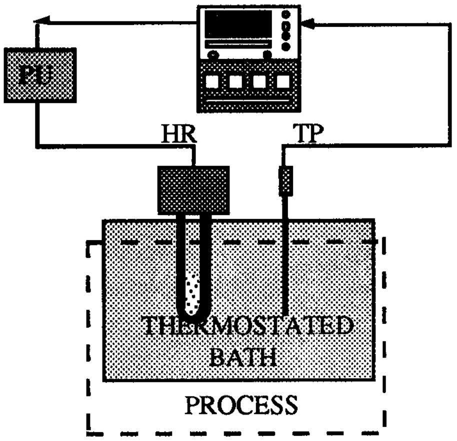

*Figure 1. Heating loop.*

0142-0453/97 $12·00 © 1997 Taylor & Francis Ltd

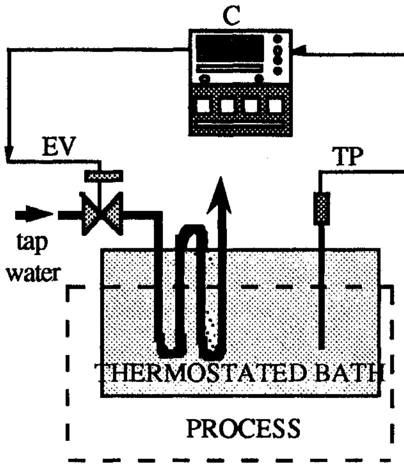

*Figure 2. Cooling loop.*

*The cooling loop*

The cooling loop consists of the same controller (C) and Pt100 probe (TP) immersed in the thermostatted bath. Tap water flows through a coil (average temperature 16 °C) when an electrovalve (EV) is opened. For crystallization processes requiring operations below 20 °C, a water/glycol mix cooled to −10 °C is used instead of tap water. A static relay is activated to allow the passage of this mix. This regulation loop is shown in figure 2. The cooling system is controlled through the 'cold' channel with a discontinuous modulated signal (0–10 V). Before using controllers without autosetting capabilities, the regulation parameters must be determined as follows:

(1) The proportional band, expressed as the variation in a percentage of the total measurement range.
(2) The integral time (in seconds) which represents the necessary time lapse before the signal output variation equals the variation caused by the proportional action.
(3) The derived time which allows the effects of the proportional action to be amplified.
(4) The modulation time.

These parameters are set during the process identification phase.

*Parameter setting*

The majority of regulation loops can be accomplished simply with an empirical adjustment of the parameters of universal controllers. Other loops are more complicated and it is necessary to know the dynamics of the process concerned. Most systems are naturally stable and, when displaced out of their equilibrium state by a perturbation or a corrective action, they tend to attain a new state of equilibrium after a certain time. This dynamic phase of the system is a characteristic function of the process.

The optimal parameter values for a PID controller depend only on the characteristics of the system to be regulated and on the controller which is being used. The system is identified in this case by determining its transfer function—the function correlating the influent value and the controlled value. Analysis of a graph showing the evolution of the controlled value over time allows the transfer function to be calculated and also, therefore, the most favourable conditions for the stability of the system, i.e. the parameters to be set on the controller. The identification of a process is the key to finding its transfer function. Identification is often a delicate task, but it is necessary to calculate the controller's actions. It is often accomplished by creating perturbations. The investigation of the system's response was conducted in this study in an open loop, using the Broida method [2].

*Implementation of the Broida method*

For system identification purposes, the controller must be set to manual mode, i.e. disconnected. The system must also be in the permanent regime, with the output and command signals, $O(s)$ and $C(s)$, constant. This is an open loop process. A perturbation is then applied to the system; the most common perturbation used is an incremental step. The command signal is incremented, and the output signal is recorded.

The transfer function for a first order system with pure delay can be expressed as follows:

$$H_s = \frac{G_s \times e^{-\theta \cdot s}}{\tau \cdot s + 1} \tag{1}$$

where $Gs$ = static gain; $\tau$ = time constant; $\theta$ = pure delay.

The theoretical Broida graph (see figure 3) (first order system with pure delay) shows the output value to be equal to 0·28 times the final variation at the time $(t_1 - \theta)$; and, at $(t_2 - \theta)$, 0·40 of the final variation:

$$O_1(t) = 0{\cdot}28 = 1 - e^{-\frac{t_1-\theta}{\tau}} \tag{2}$$

$$O_2(t) = 0{\cdot}40 = 1 - e^{-\frac{t_2-\theta}{\tau}} \tag{3}$$

These equations lead to the value of the time constant and $\theta$:

$$\tau = 5{\cdot}5(t_2 - t_1) \tag{4}$$

$$\theta = 2{\cdot}8 \cdot t_1 - 1{\cdot}8 \cdot t_2 \tag{5}$$

In most cases, it is impossible to predict the static behaviour of a process because the exact influence of the perturbations is difficult to evaluate. Generally, the

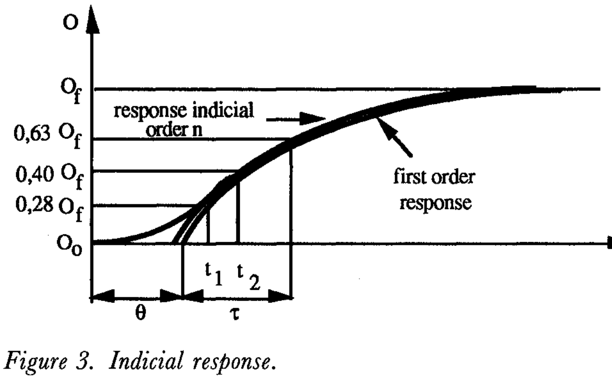

*Figure 3. Indicial response.*

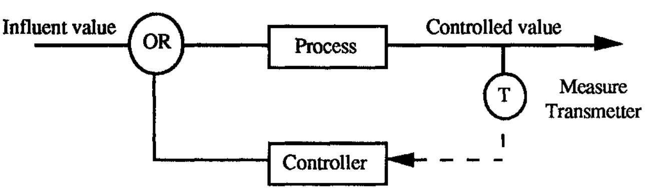

*Figure 4. Control with open loop.*

characteristics of a process are determined with an empirical method. The influent variable is changed in steps covering the full range of possible values, and a correlation is derived from observing the resulting effects on the controlled value.

### *Indicial response*

Indicial response of the system to incremental steps was studied in order to determine the types of action that a controller needs to perform. This analysis is conducted in an open loop (the controller does not react to the temperature read from the probe). The programmer is disconnected from the controller (see figure 4).

An incremental step $\Delta Y$, corresponding to a variation of the heating or cooling power of $Y$% at the controller's output, is applied. This is equivalent to sending a constant signal to the regulating device. The resulting increase ($\Delta X$) of the response signal (controlled value) is measured until the system stabilizes. Static gain is:

$$G_s = \frac{\Delta X}{E} \cdot \frac{YM}{\Delta Y} \tag{6}$$

where $E$ = total temperature range (−200·0 °C to +200·0 °C); $YM$ = total variation of the output variable; $\Delta X$ = variation of the input variable; $\Delta Y$ = variation of the output variable.

The operational parameters are shown in table 1.

### *Response of the two channels*

The temperature in the thermostatted bath is recorded, the characteristic durations $t_1$ and $t_2$ are determined from the recording of the indicial responses (see table 2). The values of the latency time, the pure delay and the static gain can be derived from $t_1$ and $t_2$ (see table 3). Table 4 shows the formulae established by Ziegler and Nichols to determine the values of the PID parameters of a controller. The proportional band $X_p$ is expressed as a percentage of the setting range. The parameters $X_p$ and $T_i$ were determined for both channels. The results are shown in table 5.

The regulation mode's choice depends on the adaptability of the system, measured by the $\tau/\theta$ ratio:

(1) If $\tau/\theta > 20$, then the regulation must be Boolean in nature.

(2) If $15{\cdot}5 < \tau/\theta < 20$, then the regulation must be proportional.

(3) If $7{\cdot}5 < \tau/\theta < 15{\cdot}5$, then the regulation must be proportional-integral.

(4) If $2{\cdot}5 < \tau/\theta < 7{\cdot}5$, then the regulation must be PID.

(5) If $\tau/\theta < 2{\cdot}5$, then a cascade regulation must be chosen.

For the 'hot' channel: $\tau/\theta = 27{\cdot}5$, the regulation must thus be of a Boolean type. For the 'cold' channel: $\tau/\theta = 15{\cdot}5$, the regulation must be PI.

As the controller requires common setting parameters for both channels (for the integral and derived time constants), the best adapted regulation was considered for this experiment to be of the type PI:

$$XP_H = 15; XP_C = 6; T_i = (720 + 414)/2 = 567; T_d = 0$$

The overlapping segment, $R$, was arbitrarily set to zero, because no examples of application to hot/cold regulation were found in the literature. The introduction of these five parameters: $XP_H$, $XP_C$, $T_i$, $T_d$ and $R$ in the controller, allowed the authors' first attempts at controlling the temperature in the reactor/crystallizer.

*Table 1. Operational parameters.*

| | *'Hot' channel* | *'Cold' channel* |
|---|---|---|
| Output variation | 0 to 100% | −100 to 0% |
| Incremental step | 10% | 10% |
| Variation of the controlled value | 15·6 °C | 20·3 °C |

*Table 2. Characteristic time values.*

| *Time* | *'Hot' channel* | *'Cold' channel* |
|---|---|---|
| $t_1$ | 2400 | 840 |
| $t_2$ | 3600 | 1230 |

*Table 3. Static gain, time constant, pure delay.*

| *Parameter* | *'Hot' channel* | *'Cold' channel* |
|---|---|---|
| $\tau$ | 5·5 · (3600 − 2400) = 6600 s | 2145 s |
| $\theta$ | 2·8 · (2400) − 1·8 · (3600) = 240 *s* | 138 s |
| *Gs* | (15·6 · 100)/(400 · 10) = 0·39 | 0·5075 |

*Table 4. Ziegler–Nichols formulae for each type of regulation ($T_i$ is the integral time constant and $T_d$ is the derived time constant).*

| | *P* | *P.I.* | *P.I.D.* |
|---|---|---|---|
| $X_p$ (%) | $\frac{\theta}{\tau} Gs \cdot 100$ | $> 1{\cdot}2 \cdot \frac{\theta}{\tau} Gs \cdot 100$ | $\geqslant 0{\cdot}8 \cdot \frac{\theta}{\tau} Gs \cdot 100$ |
| $T_i$ (min) | — | — | $2\theta$ |
| $T_d$ (s) | — | — | $0{\cdot}4\theta$ |

*Table 5. Calculated PID parameters.*

| *Parameter* | *'Hot' channel* | *'Cold' channel* |
|---|---|---|
| Proportional band (%) | 14·7 ($XP_H$) | 6·4 ($XP_C$) |
| $T_i$ (s) | 720 | 414 |

The indicial response of the system to a programmed set value was recorded. In a previous study [3], and in experiments with the calculated parameters, it was observed that the temperature in the reactor does not follow the set value and that considerable discrepancies appear when the process is run automatically; these discrepancies may reach 10 °C. To reduce these unwanted effects, it was decided to optimize the regulation parameters in this experiment.

### Optimization of the PID parameters on the simplex method

The aim here was to optimize the controller's parameters to obtain a value closer to the programmed value using the simplex method [4, 5]. Starting with an experiment already conducted in the experimental domain, $k$ other experiments are conducted to obtain a regular geometric shape called a 'simplex'. The responses for these $k+1$ other experiments are measured. The experiment with the least desirable result is discarded, and a new one is substituted, with co-ordinates obtained by calculating the symmetric point of the discarded point with respect to the centre of gravity of those remaining.

The coordinates for the starting simplex, expressed with reduced variables, are given in table 6. The reduced and real coordinates are linked as follows:

$$x_{i,j} = x_{1,j} + X_{i,j} \cdot \Delta x_j \qquad (7)$$

where $i$ ranged from 1 to $k+1$ and j ranged from 1 to $k$; $x_{i,j}$ = the real coordinate of the $j$th variable at the point $i$; $x_{1,j}$ = the real coordinate of the $j$th variable at the starting point; $X_{i,j}$ = the reduced variable of the $j$th variable at the point $i$; $\Delta x_j$ = the chosen incremental step for the $j$th variable.

Simplex evolution follows several rules:

(1) *Rule 1*: The worst point is discarded and is replaced by its symmetric point with respect to the centre of gravity of the remaining points.
(2) *Rule 2*: When a better point appears in $k+1$ consecutive simplexes, the value must not be overestimated, and it must be verified. This prevents endlessly producing a false optimal point. If a new experiment confirms the original value, the process is resumed; otherwise the faulty point is eliminated.
(3) *Rule 3*: When the evolution takes place on a ridge, i.e. the newest point is always the worst one, or when a new point cannot be added because it would be situated beyond the allowed boundaries (out of the domain), the symmetric of the second worst point is used instead. The following notation is used for the points forming the simplex:

$W$ = point with the worst response; $N$ = point with the next to the worst response; $B$ = point with the best response; $G$ = centre of gravity of the remaining points; $R$ = symmetric of point $W$ with respect to point $G$ (the Reflex). Coordinates for points $G$ and $R$ are given by the following equations:

$$X_{G,j} = \frac{1}{k}\sum_{i=1}^{k+1} X_{i,j} \text{ with } i \neq W \text{ and } j = 1 \text{ to } k \qquad (8)$$

$$X_{R,j} = X_{G,j} + (X_{G,j} - X_{W,j}) = 2X_{G,j} - X_{W,j} \qquad (9)$$

*Table 6. Coordinates for the starting simplex.*

| Vertices | $X_1$ | $X_2$ | $X_3$ | $X_4$ | $X_5$ | (where $p$ and $q$ are obtained with the following equations): |
|---|---|---|---|---|---|---|
| 1 | 0 | 0 | 0 | 0 | 0 | |
| 2 | $p$ | $q$ | $q$ | $q$ | $q$ | $p = \frac{1}{k\sqrt{2}}(\sqrt{k+1}+k-1)$ |
| 3 | $q$ | $p$ | $q$ | $q$ | $q$ | |
| 4 | $q$ | $q$ | $p$ | $q$ | $q$ | |
| 5 | $q$ | $q$ | $q$ | $p$ | $q$ | $q = \frac{1}{k\sqrt{2}}(\sqrt{k+1}-1)$ |
| 6 | $q$ | $q$ | $q$ | $q$ | $p$ | |

### *Simplex specifications*

The system's temperature being stable at 25 °C, an increment of 5 °C is applied and the temperature recorded. The response function, *FR* [6, 7] is used as an optimization criterion: this is is a weighted sum taking into account the decrease of the measure-set value deviation, but also the duration and amplitude of the oscillations:

$$FR = \sum S_i + a \cdot T_{osc} + b \cdot \sum E_i \qquad (10)$$

where $S_i$ = surface of the oscillations around the set point; $T_{osc}$ = duration of the oscillations, expressed in mm of paper ; $E_i$ = deviation between the measure and the set value.

Arbitrary values, 1 and 3, were chosen for the constant $a$ and $b$; this was to ensure that the weight of each term in the expression would be equivalent. The function was chosen to favour regulation rather than tracking. The surface is determined as follows: the surface contained between the temperature curve and the line made by the set value drawn on a fixed-density paper is cut and weighted; the weight is converted in a surface expressed in square millimeters. The object of this optimization is to minimize the *FR*.

### *Variable choice*

Five variables are likely to affect the quality of the regulation in some way:

(1) The proportional width of the 'hot' channel, expressed in %.
(2) The proportional width of the 'cold' channel, expressed in %.
(3) The integral time (expressed in seconds).
(4) The derived times (expressed in seconds).
(5) The overlapping segment of the channels (in %).

So, the system has five variables; the geometric representation of the simplex is therefore a six-vertex figure in five-dimensional space.

*Table 7. Reduced and real coordinates for the first simplex.*

| Vertex | $X_1$ | $X_2$ | $X_3$ | $X_4$ | $X_5$ | $XP_H$ | $XP_C$ | $T_i$ | $T_d$ | $R$ |
|---|---|---|---|---|---|---|---|---|---|---|
| 1 | 0·000 | 0·000 | 0·000 | 0·000 | 0·000 | 15·0 | 6·0 | 570 | 0·0 | 0·0 |
| 2 | 0·912 | 0·205 | 0·205 | 0·205 | 0·205 | 20·0 | 7·0 | 580 | 5·0 | 4·0 |
| 3 | 0·205 | 0·912 | 0·205 | 0·205 | 0·205 | 16·0 | 10·5 | 580 | 5·0 | 4.0 |
| 4 | 0·205 | 0·205 | 0·912 | 0·205 | 0·205 | 16·0 | 7·0 | 615 | 5·0 | 4·0 |
| 5 | 0·205 | 0·205 | 0·205 | 0·912 | 0·205 | 16·0 | 7·0 | 580 | 23·0 | 4·0 |
| 6 | 0·205 | 0·205 | 0·205 | 0·205 | 0·912 | 16·0 | 7·0 | 580 | 5·0 | 18·0 |

The starting coordinates used were the values of the parameters obtained with the Ziegler–Nichols identification of the system. The increment values chosen were sufficiently large to ensure significative variations in the results of each experiment:

$$\Delta XP_H = 5;\ \Delta XP_C = 5;\ \Delta T_i = 50;\ \Delta T_d = 25;\ \Delta R = 20$$

Table 7 shows the reduced and real co-ordinates for the first simplex.

*Boundaries*

The applicable domain is limited by a number of constraints. First, the proportional bands of the 'hot' and 'cold' channels must be an integer between 0% and 100%; they have to be integer numbers above 10%. Second, the integral time constant must be an integer between 0 and 1999 s. Third, the derived time constant must be between 0 and 999 s; it must be an integer above 10. Finally, the overlapping of the bands must vary between −10% and +20% of the total measurement range, in increments of 0·1%.

*Results and interpretation*

The initial simplex evolution can be traced in table 8, which shows the numerical values for all five coordinates and the value of the response function. Three values that proved useful for obtaining indications about the best moment to stop the evolution are also given:

(1) The deviation between the responses at the best and worst points: $Y_W - Y_B$.
(2) The value of the standard deviation (SD):

$$SD = \left(\frac{[k\sum Y^2 - (\sum Y)^2]}{[k(k-1)]}\right)^{0\cdot5} \quad (11)$$

(3) The value of the root-mean square deviation of the responses of the vertices from the mean response (RMS):

$$RMS = \frac{\sum(Y - \bar{Y})^2}{\sqrt{k}} \quad (12)$$

In simplex 3, the new point, 8, remains the worst one, but because it presents a better response than point 4 whose symmetric it is, it is kept in the simplex, and the symmetric from the next to worst point, 3, is taken instead. The iterations continue normally until simplex 10. At this point, when the point 12 is discarded ($Y_{12} = 330$), point 16 is obtained but eliminated because its response is poorer ($Y_{16} = 370$). The next to worst point, 15, would not be discarded because this would lead to point 6, which has already been tested. The third worst point, 7 ($Y_7 = 302$), was thus removed, and point 17 was obtained but also discarded because of its poorer response ($Y_{17} = 336$). The fourth worst point, 14, was then discarded.

In simplex 12, as the new point is the worst, it is discarded; the evolution of the simplex is then stopped. The responses have been improved from 814 to 240. The value of the three criteria has also been significantly reduced: 78 instead of 482 for the deviation between the extreme responses, 32 instead of 165 for the standard deviation and 12 instead of 62 for the root-mean square deviation.

*Optimization by the Nelder and Mead method*

This derived method uses a variable-increment simplex [8–10]. If the symmetric becomes the best of all the points of the new simplex, then the direction of the last move is considered to be the most profitable. An extension is then applied. The following cases must be distinguished:

(1) If the response $R$ is better than the response of $B$ (best point of the simplex), the simplex is extended beyond the point $R$ and the response at a new point, $E$, is measured. The responses $Y_E$ and $Y_R$ are compared; the best point replaces the point $W$ and forms the new simplex.
(2) If the response is worse than $Y_B$ but better than $Y_N$, the process resumes without changing the size of the simplex.
(3) If the response is worse than $Y_N$, two cases are distinguished: (*a*) $Y_B$ is better than $Y_W$: a contraction is applied at the side containing $R$ and point $C_R$ is added; (*b*) $Y_R$ is still worse than $Y_W$: a contraction is applied at the side containing $W$ and point $C_W$ is added.

The coordinates of the points making up expanded (or contracted) simplex are given by the formula:

$$X_{P,j} = X_{G,j} + \gamma(X_{G,j} - X_{W,j}) \quad (13)$$

where $\gamma = 2$ for $E$; $\gamma = 0\cdot5$ for $C_R$; $\gamma = -0\cdot5$ for $C_W$.

Table 8. Initial simplex evolution.

| | Nº | x1 | x2 | x3 | x4 | x5 | FR | | |
|---|---|---|---|---|---|---|---|---|---|
| Simplex n°1 | | | | | | | | | Yw-YB= |
| | 1 | 15 | 6 | 570 | 0 | 0 | 814 | W | 482 |
| | 2 | 20 | 7 | 580 | 5 | 4 | 452 | | |
| | 3 | 16 | 10 | 580 | 5 | 4 | 469 | | SD= |
| | 4 | 16 | 7 | 615 | 5 | 4 | 491 | N | 165 |
| | 5 | 16 | 7 | 580 | 25 | 4 | 419 | | RMS= |
| | 6 | 16 | 7 | 580 | 5 | 18 | 332 | B | 62 |
| Simplex n°2 | | | | | | | | | Yw-YB= |
| R/1 | 7 | 19 | 9.2 | 604 | 17 | 13.6 | 302 | B | 189 |
| | 2 | 20 | 7 | 580 | 5 | 4 | 452 | | |
| | 3 | 16 | 10 | 580 | 5 | 4 | 469 | N | SD= |
| | 4 | 16 | 7 | 615 | 5 | 4 | 491 | W | 77 |
| | 5 | 16 | 7 | 580 | 25 | 4 | 419 | | RMS= |
| | 6 | 16 | 7 | 580 | 5 | 18 | 332 | | 29 |
| Simplex n°3 | | | | | | | | | Yw-YB= |
| | 7 | 19 | 9.2 | 604 | 17 | 13.6 | 302 | B | 180 |
| | 2 | 20 | 7 | 580 | 5 | 4 | 452 | | |
| | 3 | 16 | 10 | 580 | 5 | 4 | 469 | N | SD= |
| R/4 | 8 | 19 | 9.1 | 555 | 17 | 13.4 | 482 | W | 75 |
| | 5 | 16 | 7 | 580 | 25 | 4 | 419 | | RMS= |
| | 6 | 16 | 7 | 580 | 5 | 18 | 332 | | 28 |
| Simplex n°4 | | | | | | | | | Yw-YB= |
| | 7 | 19 | 9.2 | 604 | 17 | 13.6 | 302 | B | 180 |
| | 2 | 20 | 7 | 580 | 5 | 4 | 452 | N | |
| R/3 | 9 | 20 | 5.7 | 580 | 22 | 17.2 | 436 | | SD= |
| | 8 | 19 | 9.1 | 555 | 17 | 13.4 | 482 | W | 71 |
| | 5 | 16 | 7 | 580 | 25 | 4 | 419 | | RMS= |
| | 6 | 16 | 7 | 580 | 5 | 18 | 332 | | 26 |
| Simplex n°5 | | | | | | | | | Yw-YB= |
| | 7 | 19 | 9.2 | 604 | 17 | 13.6 | 302 | B | 150 |
| | 2 | 20 | 7 | 580 | 5 | 4 | 452 | W | |
| | 9 | 20 | 5.7 | 580 | 22 | 17.2 | 436 | N | SD= |
| R/8 | 10 | 17 | 5.3 | 615 | 12 | 9.3 | 409 | | 60 |
| | 5 | 16 | 7 | 580 | 25 | 4 | 419 | | RMS= |
| | 6 | 16 | 7 | 580 | 5 | 18 | 332 | | 23 |
| Simplex n°6 | | | | | | | | | Yw-YB= |
| | 7 | 19 | 9.2 | 604 | 17 | 13.6 | 302 | | 160 |
| R/2 | 11 | 15 | 6.7 | 604 | 27 | 20 | 276 | B | |
| | 9 | 20 | 5.7 | 580 | 22 | 17.2 | 436 | W | SD= |
| | 10 | 17 | 5.3 | 615 | 12 | 9.3 | 409 | | 68 |
| | 5 | 16 | 7 | 580 | 25 | 4 | 419 | N | RMS= |
| | 6 | 16 | 7 | 580 | 5 | 18 | 332 | | 25 |

| | Nº | x1 | x2 | x3 | x4 | x5 | FR | | |
|---|---|---|---|---|---|---|---|---|---|
| Simpex n°7 | | | | | | | | | Yw-YB= |
| | 7 | 19 | 9.2 | 604 | 17 | 13.6 | 302 | | 143 |
| | 11 | 15 | 6.7 | 604 | 27 | 20 | 276 | B | |
| R/9 | 12 | 13 | 8.4 | 613 | 12 | 8.8 | 330 | | SD= |
| | 10 | 17 | 5.3 | 615 | 12 | 9.3 | 409 | N | 58 |
| | 5 | 16 | 7 | 580 | 25 | 4 | 419 | W | RMS= |
| | 6 | 16 | 7 | 580 | 5 | 18 | 332 | | 21 |
| Simplex n°8 | | | | | | | | | Yw-YB= |
| | 7 | 19 | 9.2 | 604 | 17 | 13.6 | 302 | | 149 |
| | 11 | 15 | 6.7 | 604 | 27 | 20 | 276 | | |
| | 12 | 13 | 8.4 | 613 | 12 | 8.8 | 330 | | SD= |
| | 10 | 17 | 5.3 | 615 | 12 | 9.3 | 409 | W | 53 |
| R/5 | 13 | 16 | 7.6 | 626 | 6.2 | 20 | 260 | B | RMS= |
| | 6 | 16 | 7 | 580 | 5 | 18 | 332 | N | 20 |
| Simplex n°9 | | | | | | | | | Yw-YB= |
| | 7 | 19 | 9.2 | 604 | 17 | 13.6 | 302 | | 72 |
| | 11 | 15 | 6.7 | 604 | 27 | 20 | 276 | | |
| | 12 | 13 | 8.4 | 613 | 12 | 8.8 | 330 | N | SD= |
| R/10 | 14 | 15 | 10 | 596 | 15 | 20 | 300 | | 29 |
| | 13 | 16 | 7.6 | 626 | 6,2 | 20 | 260 | B | RMS= |
| | 6 | 16 | 7 | 580 | 5 | 18 | 332 | W | 11 |
| Simplex n°10 | | | | | | | | | Yw-YB= |
| | 7 | 19 | 9.2 | 604 | 17 | 13.6 | 302 | N' | 70 |
| | 11 | 15 | 6.7 | 604 | 27 | 20 | 276 | | |
| | 12 | 13 | 8.4 | 613 | 12 | 8.8 | 330 | W | SD= |
| | 14 | 15 | 10 | 596 | 15 | 20 | 300 | N" | 26 |
| | 13 | 16 | 7.6 | 626 | 6.2 | 20 | 260 | B | RMS= |
| R/6 | 15 | 15 | 9.8 | 637 | 26 | 15 | 318 | N | 10 |
| R/12 | 16 | 19 | 8.9 | 614 | 25 | 20 | 370 | discarded | |
| R/7 | 17 | 11 | 7.8 | 626 | 17 | 20 | 336 | discarded | |
| Simplex n°11 | | | | | | | | | Yw-YB= |
| | 7 | 19 | 9.2 | 604 | 17 | 13.6 | 302 | | 90 |
| | 11 | 15 | 6.7 | 604 | 27 | 20 | 276 | | |
| | 12 | 13 | 8.4 | 613 | 12 | 8.8 | 330 | W | SD= |
| R/14 | 18 | 16 | 6.7 | 638 | 20 | 11 | 240 | B | 35 |
| | 13 | 16 | 7.6 | 626 | 6.2 | 20 | 260 | | RMS= |
| | 15 | 15 | 9.8 | 637 | 26 | 15 | 318 | N | 13 |
| Simplex n°12 | | | | | | | | | Yw-YB= |
| | 7 | 19 | 9.2 | 604 | 17 | 13.6 | 302 | N | 78 |
| | 11 | 15 | 6.7 | 604 | 27 | 20 | 276 | | |
| R/12 | **19** | **19** | **7.6** | **631** | **26** | **20** | **240** | **B** | SD= |
| | **18** | **16** | **6.7** | **638** | **20** | **11** | **240** | **B** | 32 |
| | 13 | 16 | 7.6 | 626 | 6.2 | 20 | 260 | | RMS= |
| | 15 | 15 | 9.8 | 637 | 26 | 15 | 318 | W | 12 |
| R/15 | 20 | 19 | 5.3 | 604 | 12 | 19 | 340 | W | |

*Table 9. Evolution following the Nelder and Mead method.*

| | N° | x1 | x2 | x3 | x4 | x5 | FR | | |
|---|---|---|---|---|---|---|---|---|---|
| Simplex n°1 | | | | | | | | | Yw-YB= |
| | 1 | 15 | 6 | 570 | 0 | 0 | 814 | W | 482 |
| | 2 | 20 | 7 | 580 | 5 | 4 | 452 | | |
| | 3 | 16 | 10 | 580 | 5 | 4 | 469 | | SD = |
| | 4 | 16 | 7 | 615 | 5 | 4 | 491 | N | 165 |
| | 5 | 16 | 7 | 580 | 23 | 4 | 419 | | RMS = |
| | 6 | 16 | 7 | 580 | 5 | 18 | 332 | B | 62 |
| R/1 | 7 | 19 | 9.2 | 604 | 17 | 13.6 | 302 | | |
| E /1 | 8 | 20 | 11 | 621 | 26 | 20 | 240 | | |
| Simplex n°2 | | | | | | | | | Yw-YB= |
| | 8 | 20 | 11 | 621 | 26 | 20 | 240 | B | 251 |
| | 2 | 20 | 7 | 580 | 5 | 4 | 452 | | |
| | 3 | 16 | 10 | 580 | 5 | 4 | 469 | N | SD = |
| | 4 | 16 | 7 | 615 | 5 | 4 | 491 | W | 96 |
| | 5 | 16 | 7 | 580 | 23 | 4 | 419 | | RMS = |
| | 6 | 16 | 7 | 580 | 5 | 18 | 332 | | 36 |
| Simplex n°3 | | | | | | | | | Yw-YB= |
| | 8 | 20 | 11 | 621 | 26 | 20 | 240 | B | 229 |
| | 2 | 20 | 7 | 580 | 5 | 4 | 452 | N | |
| | 3 | 16 | 10 | 580 | 5 | 4 | 469 | W | SD = |
| R/4 | 9 | 19 | 9.8 | 561 | 21 | 16 | 308 | | 91 |
| | 5 | 16 | 7 | 580 | 23 | 4 | 419 | | RMS = |
| | 6 | 16 | 7 | 580 | 5 | 18 | 332 | | 34 |
| Simplex n°4 | | | | | | | | | Yw-YB= |
| | 8 | 20 | 11 | 621 | 26 | 20 | 240 | B | 212 |
| | 2 | 20 | 7 | 580 | 5 | 4 | 452 | W | |
| R/3 | 10 | 20 | 6.7 | 589 | 27 | 20 | 293 | | SD = |
| | 9 | 19 | 9.8 | 561 | 21 | 16 | 308 | | 30 |
| | 5 | 16 | 7 | 580 | 23 | 4 | 419 | N | RMS = |
| | 6 | 16 | 7 | 580 | 5 | 18 | 332 | | 80 |
| Simplex n°5 | | | | | | | | | Yw-YB= |
| | 8 | 20 | 11 | 621 | 26 | 20 | 240 | B | 179 |
| R/2 | 11 | 16 | 9.6 | 592 | 36 | 20 | 276 | | |
| | 10 | 20 | 6.7 | 589 | 27 | 20 | 293 | | SD = |
| | 9 | 19 | 9.8 | 561 | 21 | 16 | 308 | | 61 |
| | 5 | 16 | 7 | 580 | 23 | 4 | 419 | W | RMS = |
| | 6 | 16 | 7 | 580 | 5 | 18 | 332 | N | 23 |
| R/5 | 12 | 20 | 11 | 597 | 23 | 20 | 370 | | |
| C/12 | 13 | 19 | 9.9 | 593 | 23 | 20 | 288 | | |
| Simplex n°6 | | | | | | | | | Yw-YB= |
| | 8 | 20 | 11 | 621 | 26 | 20 | 240 | B | 92 |
| R/2 | 11 | 16 | 9.6 | 592 | 36 | 20 | 276 | | |
| | 10 | 20 | 6.7 | 589 | 27 | 20 | 293 | | SD = |
| | 9 | 19 | 9.8 | 561 | 21 | 16 | 308 | N | 31 |
| | 13 | 19 | 9.9 | 593 | 23 | 20 | 288 | | RMS = |
| | 6 | 16 | 7 | 580 | 5 | 18 | 332 | W | 12 |
| Simplex n°7 | | | | | | | | | Yw-YB= |
| | 8 | **20** | **11** | **621** | **26** | **20** | **240** | **B** | 68 |
| | 11 | 16 | 9.6 | 592 | 36 | 20 | 276 | | |
| | 10 | 20 | 6.7 | 589 | 27 | 20 | 293 | | SD = |
| | 9 | 19 | 9.8 | 561 | 21 | 16 | 308 | W | 25 |
| | 13 | 19 | 9.9 | 593 | 23 | 20 | 288 | | RMS = |
| R/6 | 14 | 22 | 12 | 602 | 48 | 20 | 305 | N | 9 |
| R/9 | 15 | 20 | 9.9 | 638 | 43 | 20 | 338 | Discarded | |

*Table 10. Evolution following the Hendrix method.*

| | N° | x1 | x2 | x3 | x4 | x5 | FR | | |
|---|---|---|---|---|---|---|---|---|---|
| Simplex n°1 | | | | | | | | | Yw-YB= |
| | 1 | 15 | 6 | 570 | 0 | 0 | 814 | W | 482 |
| | 2 | 20 | 7 | 580 | 5 | 4 | 452 | B | |
| | 3 | 16 | 10 | 580 | 5 | 4 | 469 | B | SD = |
| | 4 | 16 | 7 | 615 | 5 | 4 | 491 | B | 165 |
| | 5 | 16 | 7 | 580 | 23 | 4 | 419 | B | RMS = |
| | **6** | **16** | **7** | **580** | **5** | **18** | **332** | B | 62 |
| Simplex n°2 | | | | | | | | | Yw-YB= |
| R/1 | **7** | **19** | **9.2** | **604** | **17** | **13.6** | **302** | B | 189 |
| | 2 | 20 | 7 | 580 | 5 | 4 | 452 | W | |
| | 3 | 16 | 10 | 580 | 5 | 4 | 469 | W | SD = |
| | 4 | 16 | 7 | 615 | 5 | 4 | 491 | W | 77 |
| | 5 | 16 | 7 | 580 | 23 | 4 | 419 | W | RMS = |
| | 6 | 16 | 7 | 580 | 5 | 18 | 332 | B | 29 |
| Simplex n°3 | | | | | | | | | Yw-YB= |
| | 7 | 19 | 9.2 | 604 | 17 | 13.6 | 302 | B | 307 |
| R/2 | 8 | 15 | 9.2 | 604 | 17 | 20 | 293 | B | |
| R/3 | 9 | 19 | 6.2 | 604 | 17 | 20 | 592 | W | SD = |
| R/4 | 10 | 19 | 9.2 | 569 | 17 | 20 | 360 | B | 117 |
| R/5 | **11** | **19** | **9.2** | **604** | **0** | **20** | **285** | B | RMS = |
| | 6 | 16 | 7 | 580 | 5 | 18 | 332 | B | 43 |
| Simplex n°4 | | | | | | | | | Yw-YB= |
| | 7 | 19 | 9.2 | 604 | 17 | 13.6 | 302 | B | 75 |
| | 8 | 15 | 9.2 | 604 | 17 | 20 | 293 | B | |
| R/9 | 12 | 16 | 11 | 580 | 5.4 | 16.6 | 303 | B | SD = |
| | 10 | 19 | 9.2 | 569 | 17 | 20 | 360 | W | 28 |
| | **11** | **19** | **9.2** | **604** | **0** | **20** | **285** | B | RMS = |
| | 6 | 16 | 7 | 580 | 5 | 18 | 332 | B | 11 |
| Simplex n°5 | | | | | | | | | Yw-YB= |
| | 7 | 19 | 9.2 | 604 | 17 | 13.6 | 302 | B | 47 |
| | 8 | 15 | 9.2 | 604 | 17 | 20 | 293 | B | |
| | 12 | 16 | 11 | 580 | 5.4 | 16.6 | 303 | B | SD = |
| R/10 | 13 | 15 | 9 | 620 | 1 | 15.3 | 330 | W | 19 |
| | **11** | **19** | **9.2** | **604** | **0** | **20** | **285** | B | RMS = |
| | 6 | 16 | 7 | 580 | 5 | 18 | 332 | W | 7 |
| Simplex n°6 | | | | | | | | | Yw-YB= |
| | 7 | 19 | 9.2 | 604 | 17 | 13.6 | 302 | B | 158 |
| | 8 | 15 | 9.2 | 604 | 17 | 20 | 293 | B | |
| | 12 | 16 | 11 | 580 | 5.4 | 16.6 | 303 | B | SD = |
| R/13 | 14 | 20 | 10 | 576 | 19 | 19.8 | 398 | W | 52 |
| | 11 | 19 | 9.2 | 604 | 0 | 20 | 285 | B | RMS = |
| R/6 | **15** | **19** | **12** | **616** | **15** | **17.1** | **240** | B | 19 |
| Simplex n°7 | | | | | | | | | Yw-YB= |
| | 7 | 19 | 9.2 | 604 | 17 | 13.6 | 302 | B | 122 |
| | 8 | 15 | 9.2 | 604 | 17 | 20 | 293 | B | |
| | 12 | 16 | 11 | 580 | 5.4 | 16.6 | 303 | B | SD = |
| | 16 | 15 | 10 | 627 | 2.8 | 15.1 | 362 | W | 39 |
| | 11 | 19 | 9.2 | 604 | 0 | 20 | 285 | B | RMS = |
| R/6 | **15** | **19** | **12** | **616** | **15** | **17.1** | **240** | B | 15 |

For each simplex, the best vertex is expressed in bold

*Results and interpretation*

Table 9 shows the evolution of the original simplex, with numerical values for the five sets of coordinates, and the value of the response function 'FR', including data about the three ending criteria. In order to build the second simplex, as the response of the symmetric point is the best of all, an expansion is applied, and retained. No further size alteration occurs until the fifth simplex.

The sixth simplex was built from a contraction, as the symmetric point for point 5 ($Y_5 = 419$) led to a better response ($Y_{12} = 370$), but it was the worst of this new simplex. Thus, a contraction was applied on point 12's side.

In the seventh simplex, the symmetric point for point 9 ($Y_9 = 308$) was found not to be an improvement over the previous ones ($Y_{15} = 338$). The evolution of the simplex was discontinued at this point because: as with the initial method, the response was improved from 814 to 240 for the best point of the last simplex; the variation between the points of the last simplex is of the same order of magnitude: 68 (instead of 78); the SD has the same value: 31; the RMS was lower: 9 (instead of 12). So, 15 experiments were enough to obtain comparable results. The best point was obtained in the first expansion in the second simplex.

*Optimization by the multi-move method*

This method [11] divides the experiments into groups of good and bad points rather than a single bad point and the rest. This could allow for a planning and lead to a reduced number of experiments.

*Results and interpretation*

Table 10 gives the data used in the evolution of the original simplex. In the first basic simplex, the Hendrix method requires no alteration because the group of bad points only comprises point 1. In the second simplex, however, the group of bad points includes points 2, 3, 4 and 5 (see table 11).

The most important variation between the mean values is 140·8. Points 2, 3, 4 and 5 were therefore discarded. This process was repeated until point 16 was reached, with a response of $Y_{16} = 362$. If two groups of points were defined, then point 16 was the group of bad points. Consequently, point 16 was discarded; but then point 14 resulted. The simplex evolution was ended at that point because the same best response $Y_{15} = 240$ was obtained, although the variation between the points of the last simplex was higher (122 instead of 78). The SD and RMS were very close (39 for 32 and 15 for 12).

The results of this method, in the third simplex, were better than the original method because its 10th simplex had responses varying between 370 ($Y_{16}$) and 260 ($Y_{13}$).

*Table 11. Determination of bad and good groups of points.*

| *Good points* | *Bad points* | *Average of good FR* | *Average of bad FR* | *Variation of the mean values* |
|---|---|---|---|---|
| 7 | 4, 3, 2, 5, 6 | 302 | 432, 6 | 130, 6 |
| **7, 6** | **4, 3, 2, 5** | **317** | **457, 8** | **140, 8** |
| 7, 6, 5 | 4, 3, 2 | 351 | 470, 7 | 119, 7 |
| 7, 6, 5, 2 | 4, 3 | 369 | 480 | 111 |
| 7, 6, 5, 2, 3 | 4 | 394 | 491 | 97 |

*Weighted centroid method (WCM)*

This method moves away from the worst point towards the best one [12]. In order to achieve this the determination of the symmetric point takes into account a weighted centre of gravity (the weight of each point being a factor determined from the response at the point and the worst response). The co-ordinates for the new, weighted centre are:

$$X_{G_{W,j}} = \frac{\sum(X_{i,j}(Y_i - Y_W))}{\sum(Y_i - Y_W)} \tag{14}$$

To avoid excessive degeneration of the simplex, the authors recommend limiting the value of $\gamma$, the ratio between the distance from the centre of gravity $G$ to the point $G_W$ and the distance from $G$ to the best point $B$.

$$\gamma = \|G_W - G\| / \|B - G\|$$

$$\gamma = \left[\sum (X_{G_{W,j}} - X_{G,j})^2\right]^{1/2} \Big/ \left[\sum (X_{B,j} - X_{G,j})^2\right]^{1/2} \tag{15}$$

$\gamma$ ranges from 0 to 1. The highest advisable value $\delta$ for this ratio is 0·3. If $\gamma$ is lower than $\delta$ or equal to it, the point $G_W$ is retained; otherwise, it is replaced by $G_C$, the revised centre of gravity, whose coordinates are given by [13]:

$$X_{G_{C,j}} = X_{G,j} + \delta(X_{B,j} - X_{G,j}) \tag{16}$$

$G_C$, located between $G$ and $B$, is not necessarily between $G$ and $G_W$ (unless only two variables are used for the optimization). For example, for a three-variable optimization, $G_C$ is situated on an arc of a circle whose centre is $G$. In order to keep both the line of highest increase and the ponderation, the coordinates of point $G'_C$ are determined from:

$$X_{G'_{C,j}} = X_{G,j} + \delta/\gamma(X_{G_{W,j}} - X_{G,j}) \tag{17}$$

The distance $\|G - G'_C\|$ is equal to $\|G - G_C\|$, and the point $G'_C$ is situated between $G$ and $G_W$.

*Results and interpretation*

Table 12 shows the evolution of the initial simplex. With the second simplex, because the $\delta$ ratio was higher than the limit 0·3, $G'_C$ was used as the centre of gravity. The evolution continued to the seventh simplex, where the symmetric point could no longer be considered because its response was much higher. The process ended there, with responses ranging from $Y_{13} = 170$ and $Y_9 = 275$,

*Table 12. Evolution following weighted centroid method.*

| N° | x1 | x2 | x3 | x4 | x5 | FR | | |
|---|---|---|---|---|---|---|---|---|
| Simplex n°1 | | | | | | | | Yw-YB = |
| 1 | 15 | 6 | 570 | 0 | 0 | 814 | W | 482 |
| 2 | 20 | 7 | 580 | 5 | 4 | 452 | | |
| 3 | 16 | 10 | 580 | 5 | 4 | 469 | | SD = |
| 4 | 16 | 7 | 615 | 5 | 4 | 491 | | 165 |
| 5 | 16 | 7 | 580 | 23 | 4 | 419 | | RMS = |
| 6 | 16 | 7 | 580 | 5 | 18 | 332 | B | 62 |
| Gw | 17 | 7.5 | 586 | 8.7 | 7.5 | | | γ=0.1 |
| Simplex n°2 | | | | | | | | Yw-YB = |
| R/1 7 | 19 | 9 | 602 | 17 | 15 | 240 | B | 251 |
| 2 | 20 | 7 | 580 | 5 | 4 | 452 | | |
| 3 | 16 | 10 | 580 | 5 | 4 | 469 | | SD = |
| 4 | 16 | 7 | 615 | 5 | 4 | 491 | W | 96 |
| 5 | 16 | 7 | 580 | 23 | 4 | 419 | | RMS = |
| 6 | 16 | 7 | 580 | 5 | 18 | 332 | | 36 |
| G'c | 18 | 8 | 589 | 12 | 12.3 | | | γ=0.43 |
| Simplex n°3 | | | | | | | | Yw-YB = |
| 7 | 19 | 9 | 602 | 17 | 15 | 240 | B | 229 |
| 2 | 20 | 7 | 580 | 5 | 4 | 452 | | |
| 3 | 16 | 10 | 580 | 5 | 4 | 469 | W | SD = |
| R/4 8 | 20 | 9 | 561(3)* | 19 | 20 | 358 | | 86 |
| 5 | 16 | 7 | 580 | 23 | 4 | 419 | | RMS = |
| 6 | 16 | 7 | 580 | 5 | 18 | 332 | | 32 |
| G'c | 18 | 8.2 | 585 | 14 | 14.8 | | | γ=0.39 |
| Simplex n°4 | | | | | | | | Yw-YB = |
| 7 | 19 | 9 | 602 | 17 | 15 | 240 | B | 212 |
| 2 | 20 | 7 | 580 | 5 | 4 | 452 | W | |
| R/3 9 | 20 | 6.4 | 590 | 21(3) | 20 | 275 | | SD = |
| 8 | 20 | 9 | 561 | 19 | 20 | 358 | | 81 |
| 5 | 16 | 7 | 580 | 23 | 4 | 419 | | RMS = |
| 6 | 16 | 7 | 580 | 5 | 18 | 332 | | 30 |
| G'c | 19 | 7.8 | 586 | 16 | 17 | | | γ=0.43 |
| Simplex n°5 | | | | | | | | Yw-YB = |
| 7 | 19 | 9 | 602 | 17 | 15 | 240 | | 205 |
| R/2 10 | 18 | 8.5 | 590(2) | 27 | 20 | 214 | B | |
| 9 | 20 | 6.4 | 590 | 21 | 20 | 275 | | SD = |
| 8 | 20 | 9 | 561 | 19 | 20 | 358 | | 77 |
| 5 | 16 | 7 | 580 | 23 | 4 | 419 | W | RMS = |
| 6 | 16 | 7 | 580 | 5 | 18 | 332 | | 29 |
| Gw | 19 | 8.1 | 589 | 19 | 18 | | | γ=0.28 |
| Simplex n°6 | | | | | | | | Yw-YB = |
| 7 | 19 | 9 | 602 | 17 | 15 | 240 | | 144 |
| 10 | 18 | 8.5 | 590 | 27 | 20 | 214 | B | |
| 9 | 20 | 6.4 | 590 | 21 | 20 | 275 | | SD = |
| 8 | 20 | 9 | 561 | 19 | 20 | 358 | W | 55 |
| R/5 11 | 22 | 9.2 | 598 | 15 | 20 | 265 | | RMS = |
| 6 | 16 | 7 | 580 | 5 | 18 | 332 | | 20 |
| G'c | 19 | 8.3 | 594 | 19 | 18.5 | | | γ=0.32 |
| Simplex n°7 | | | | | | | | Yw-YB = |
| 7 | 19 | 9 | 602 | 17 | 15 | 240 | | 147 |
| 10 | 18 | 8.5 | 590 | 27 | 20 | 214 | | |
| 9 | 20 | 6.4 | 590 | 21 | 20 | 275 | | SD = |
| R/8 12 | 18 | 7.6 | 627 | 19 | 17 | 185 | B | 51 |
| 11 | 22 | 9.2 | 598 | 15 | 20 | 265 | | RMS = |
| 6 | 16 | 7 | 580 | 5 | 18 | 332 | W | 19 |
| Gw | 19 | 8.2 | 605 | 20 | 18.2 | | | γ=0.06 |
| Simplex n°8 | | | | | | | | Yw-YB = |
| 7 | 19 | 9 | 602 | 17 | 15 | 240 | | 105 |
| 10 | 18 | 8.5 | 590 | 27 | 20 | 214 | | |
| 9 | 20 | 6.4 | 590 | 21 | 20 | 275 | W | SD = |
| 12 | 18 | 7.6 | 627 | 19 | 17 | 185 | | 43 |
| 11 | 22 | 9.2 | 598 | 15 | 20 | 265 | | RMS = |
| **R/6 13** | **22** | **9.4** | **630** | **35** | **18.4** | **170** | B | 16 |
| Gw | 20 | 8.7 | 617 | 26 | 18 | | | γ=0.1 |
| R/9 | 20 | 11 | 644 | 31 | 16 | 370 | | |

* values between brackets give calculated conditions with four experiments producing responses of 240 or less (the best response obtained with the other methods).

Figures 4 to 7 show the evolution of the three response criteria for each method with regard to each simplex. Figure 8 shows the evolution of the best responses; the weighted centroid method is clearly the best one.

### Conclusion

A hot/cold controller order was used to regulate crystallization operations. The system was identified with a common method, the Broida method, and the parameters were obtained with the Ziegler–Nichols method. The paper shows that this empirical method will only allow a qualitative approach to regulation and that, in some instances, the parameters obtained are unreliable and therefore cannot be used to cancel variations between the set point and the actual values.

Optimization methods were used to determine the regulation parameters and solve this identification problem. These methods are the simplex approach and its derivatives (the Nelder and Mead method, multi-move method and the weighted centroid method). The best adapted was the weighted centroid method, which produced a very good response at the 13th point. The other methods yielded less satisfactory responses (the best response obtained in the others being 240). However, the coordinates for the points with this response were not identical;

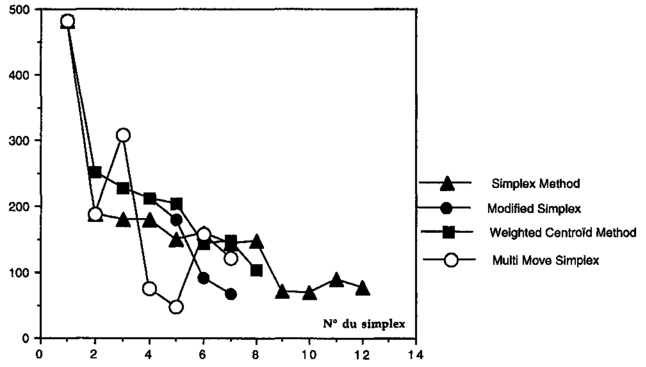

*Figure 5. Difference between extreme responses versus simplex number.*

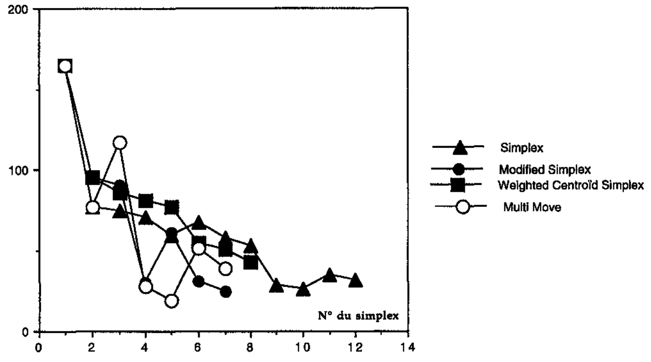

*Figure 6. Deviation standard versus simplex number.*

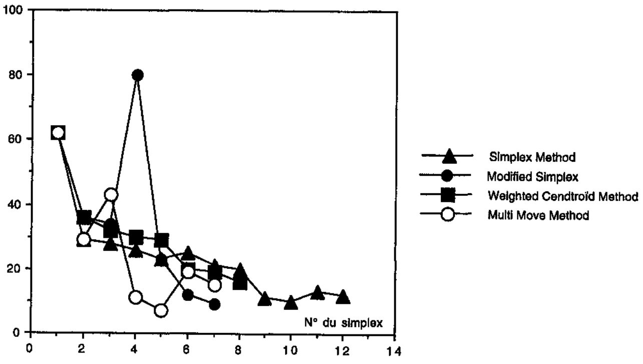

*Figure 7. Root-mean square deviation versus simplex number.*

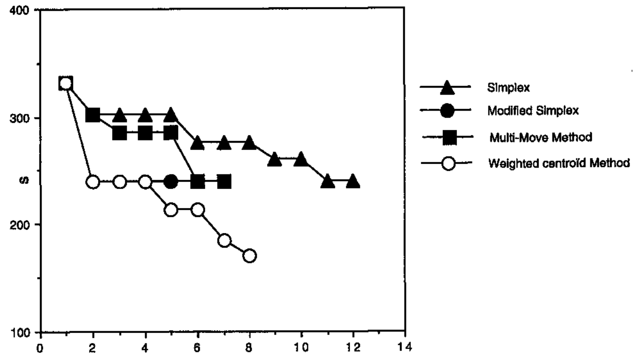

*Figure 8. Evolution of the best responses.*

this value being a compromise between surface, delay before stabilization and the variation between the set value and the actual value. So, although both points 18 and 19 have the same response at 240, point 19 is better with the simplex method. It is also better than point 15 with multi-move and point 8 with Nelder-Mead, because the temperature with the parameters at point 19 never rises above 30·7 °C, while the other points lead to temperatures above 31 °C. The most desirable characteristic for this regulation is a low deviation from the set value even if this implies longer stabilization delays. Even when the temperature is stable, deviations in the order of 0·2 °C still occur.

The weighted centroid method produced good temperature regulation in a very short time. The PID parameters obtained were quite different from the parameters calculated. The Broida method is relatively unrefined and should be used in conjunction with an optimization method. The optimized PID parameters were used in the experiments and have given satisfactory results. If the set value is 30 °C, the deviation is about 0.5 °C; it rises to about 1 °C for a set value of 60 °C.

### References

1. Caron, M., Martin Moreno, C., Bondiou, J-C., Bourgogne J-P., Porte, C. and Delacroix, A. *Bull. Soc. Chim. Fr.*, I (1991), 684.
2. Broïda, V. *Automatisme* (Tome XIV, n°3, 1969), 105
3. Martin Moreno. C., Thesis, University Pierre et Marie Curie, Paris (1989).
4. Spendley, W., Hext, G. R. and Himsworth, F. R., *Technometrics*, 4 (1962), 4, 441.

5. Porte, C., Debreuille, W. and Delacroix, A., *l'Actualité Chimique* (1984), 45.
6. Carot, S., Thesis, University Pierre et Marie Curie, Paris (1994).
7. Berridge, J. C., *Journal of Chromatography*, **244** (1982), 1.
8. Nelder, J. A. and Mead, R. *The Computer Journal*, **7** (1965), 307.
9. Porte, C., Debreuille W. and Delacroix, A., *L'Actualité Chimique* (1986), 1.
10. Morgan, E., and Burton, K. W., *Chemometrics and Intelligent Laboratory Systems*, **7** (1990), 209.
11. Hendrix, C., *Chemtech* (1980), 488.
12. Barry Ryan, P., Barr, R. L. and Todd, H. D., *Analytical Chemistry*, **52** (1980), 1460.
13. Porte, C., Debreuille, W. and Delacroix, A., *l'Actualité Chimique* (1987), 233.

